# In Silico Design of Molecular Precursors for Solution-Processable Organic Semiconductors: Structure and Electronic Properties of Sym-Azathio Circulenes

**DOI:** 10.1021/acsomega.6c05036

**Published:** 2026-09-01

**Authors:** Mosè Casalegno, Alessandro Genoni

**Affiliations:** Department of Chemistry, Materials and Chemical Engineering “Giulio Natta”, 18981 Politecnico di Milano, Via Bassini 6, Milano 20133, Italy

## Abstract

Thiophenyl circulenes, known as sulflowers, are considered potentially valuable candidates for the development of organic electronic devices. However, their usefulness as semiconducting materials is limited by their poor solubility in organic solvents. In this work, we attempt to overcome this limitation by partially replacing sulfur with nitrogen, which can be easily functionalized. The resulting compounds, namely sym-azathio circulenes, are characterized computationally. The values of selected structural and electronic descriptors compare satisfactorily with those of [8]­sulflower, showing similar trends. In particular, our ab initio calculations suggest that sym-azathio[5]­circulene preferentially behaves as a p-type semiconductor. Molecular dynamics simulations, performed on its otcyl derivative in solution and in the bulk, support the conclusion that sym-azathio circulenes may potentially provide molecular precursors for the development of solution-processable semiconductors.

## Graphical abstract

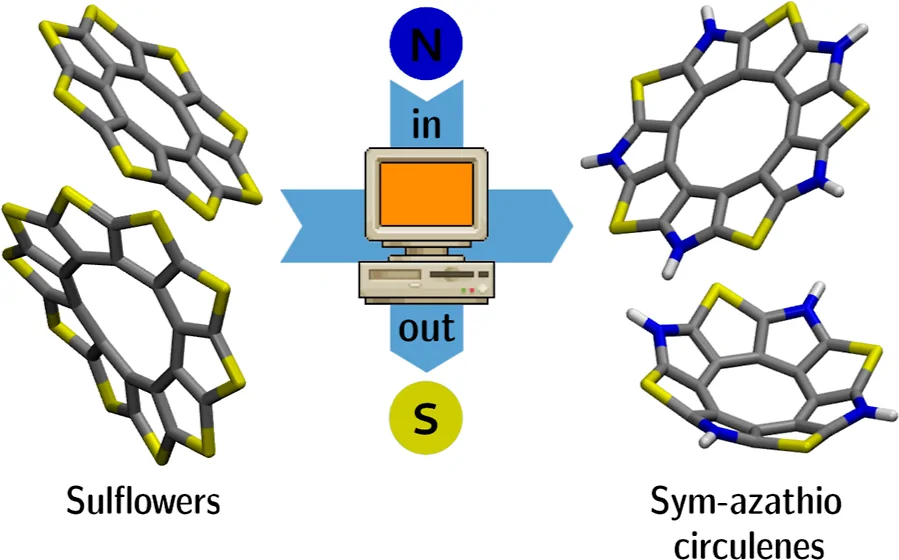

## 1 Introduction

Thiophenyl circulenes, also known as sulflowers,^1,2^ represent a family of sulfur-containing aromatic compounds, characterized by a variable number of annulated thiophene rings, with general molecular formula (C_2_S)_*n*_. Due to their extended π-conjugation and the possibility of establishing strong intermolecular interactions in the bulk,^3^ sulflowers are recognized as potentially valuable candidates in organic electronic applications.

Octathio­[8]­circulene (C_16_S_8_ [8]­sulflower, or SU8 in Figure 1), is the most extensively studied representative of this class of compounds. It is characterized by a planar structure and tightly packed molecules in the only solved crystal phase.^2^ The short intermolecular separation between neighboring molecules suggests strong π-stacking interactions. Such interactions can be expected to have a positive impact on the charge transport properties in the crystal phase,^4,5^ but limit SU8 solubility in common organic solvents.^1^ Additional reports have further questioned the usefulness of this material in electronic applications. Joo and co-workers^6^ have shown that hole mobility in SU8 crystals may not be exceptional as expected. Meanwhile, as reported by Dadvand et al.,^7^ the effective hole mobility of SU8 in thin films may be negatively affected by the growth of randomly oriented aggregates, promoted by the unfavorable balance between molecule–surface and molecule–molecule interactions.

**Figure 1:**
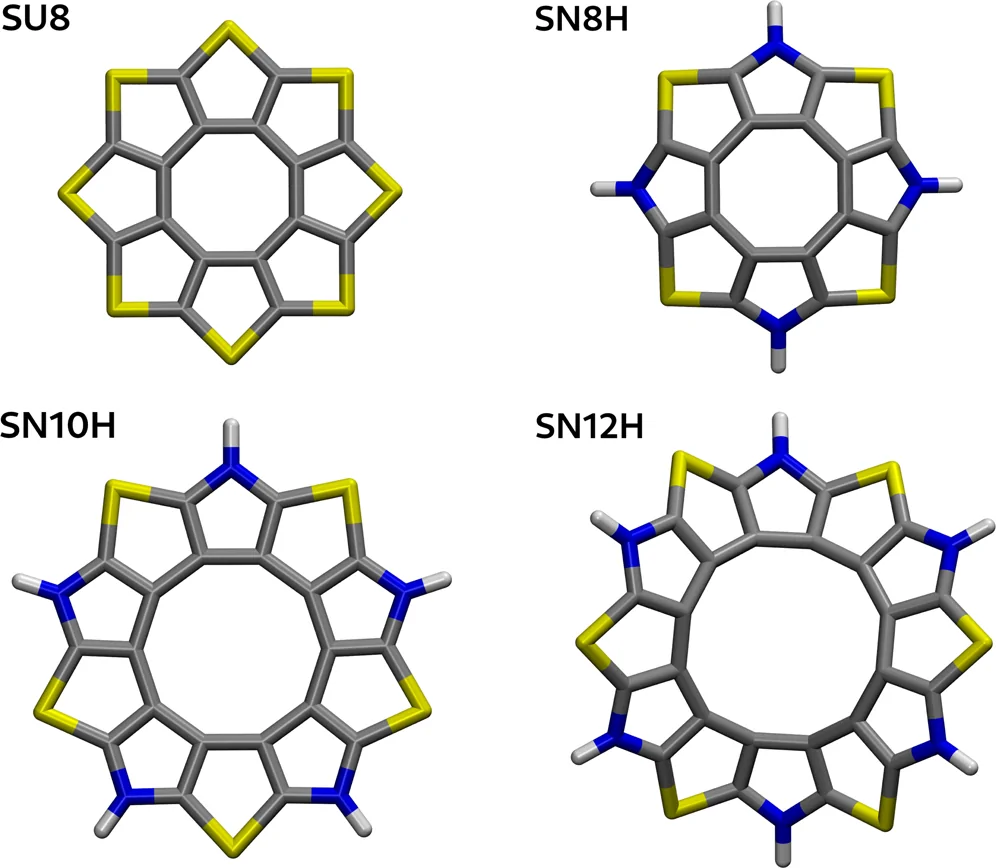
Optimized geometries of SU8, SN8H, SN10H, and SN12H (S, yellow; C, gray; N, blue; H, white), as obtained from geometry optimizations performed at B3LYP-D3BJ/6-31G** level.

The replacement of sulfur with other heteroatoms, such as selenium,^7–9^ oxygen,^10^ and nitrogen^4^ has proven effective in tuning sulflowers electronic and optical properties. However, also such compounds are plagued by low processability,^7^ plausibly due to the lack of peripheral substituents.

Parallel lines of research have focused on the possibility of synthesizing substituted hetero circulenes with tunable solubility.^11–14^ Significant work have been performed on planar molecular cores, such as hexabenzocoronenes^15,16^ and phthalocyanines,^17,18^ showing that alkyl substitution improves processability, but significantly impacts crystal packing and charge transport efficiency. The limiting case is represented by the compounds forming columnar mesophases.^19^ While these one-dimensional channels can offer high charge mobility, they are inherently more vulnerable to packing defects than two-dimensional networks. Furthermore, although structurally related to sulflowers, the presence of benzene units in these compounds suggests somewhat different electronic properties.

The above considerations raise a question whether solution-processable compounds with a sulflowers-like “all-heterole” aromatic structure can be developed. To address this problem, in this work, we considered the replacement of sulfur with nitrogen in the form of an amino NH groups. Such a chemical modification offers the 2-fold advantage of preserving the aromatic character of the core and of simultaneously introducing units suitable for further functionalization.

Instead of a full replacement of sulfur with nitrogen, which has already been tested in silico for SU8,^4^ we considered only a partial replacement with alternate exchange of sulfur and nitrogen atoms. This led to a novel class of materials, the sym-azathio­[*m*]­circulenes with general formula (C_4_SNH)_*m*_, which have been studied computationally. In particular, our computational investigation focused on some representatives of this class of compounds, corresponding to *m* = 4, 5, and 6, hereafter abbreviated as SN8H, SN10H, and SN12H. The octyl derivative of SN10H, SN10O hereafter, was also considered as a candidate solution-processable semiconductor.

The paper is organized as follows: in Section 2 materials and methods used in this investigation are described, with particular emphasis on ab initio calculations, structural descriptors adopted for the comparison of the obtained geometries, and on the details of the performed molecular dynamics simulations; Section 3 is dedicated to the analysis and discussion of the obtained results; finally, in Section 4, general conclusions are drawn and future research perspectives are briefly outlined.

## 2 Materials and Methods

### 2.1 Ab Initio Calculations

The molecular models of sym-azathio circulenes were designed through Discovery Studio Visualizer,^20^ starting from sulflower structures available in the literature.^21^ All ab initio calculations on monomers (SU8, SN8H, SN10H, and SN12H) were mainly performed at two DFT levels using functionals B3LYP-D3BJ (i.e., B3LYP functional with Grimme’s D3 dispersion corrections and Becke–Johnson damping) and M06-2X with basis set 6-31G**. Additional calculations have been carried out replacing M06-2X with other functionals of the same family, i.e. M06, M06-L, and M06-HF. Furthermore, all computations on the monomers have been repeated by also exploiting the 6-31+G** basis set. The results of all these additional calculations are reported in the Supporting Information and will be discussed below.

For SU8 and SN10H we evaluated both intra- and intermolecular charge transport properties. The intramolecular electron conductance was estimated using the orbital rule proposed by Yoshizawa.^22–24^ In particular, to account for the contributions of the HOMO, LUMO, and their degenerate or quasi-degenerate molecular orbitals (detailed below), the zeroth-order Green’s function between atomic sites *r* and *s* was calculated as follows

1 $$G_{rs}^{0}=\frac{C_{r,HOMO}C_{s,HOMO}^{*}}{E_{F}-\epsilon_{HOMO}\pm \eta i}+\frac{C_{r,LUMO}C_{s,LUMO}^{*}}{E_{F}-\epsilon_{LUMO}\pm \eta i}$$

Here, *E*_F_ denotes the Fermi energy, and *C*_*r*(*s*),HOMO(LUMO)_ represents the molecular orbital expansion coefficients for the HOMO (or LUMO) at site *r* (or *s*). The infinitesimal parameter η relates the imaginary part of the Green’s function to the local density of states; however, the imaginary term η*i* was neglected during the actual calculations. To calculate $G_{rs}^{0}$ , the molecular geometries, optimized at the B3LYP-D3BJ/6-31G** and M06-2X/6-31G** levels, were first aligned with the YZ plane using an in-house program. For SN10H, the hydrogen atoms were neglected to improve the alignment. Afterward, the π-orbital coefficients were extracted from the output of the DFT-NBO calculations using the Multiwfn software package.^25,26^

The charge transfer integrals for the SU8 and SN10H molecular dimers were calculated using the method developed by Valeev et al.,^27,28^ which evaluates the coupling between the HOMOs (or LUMOs) of the individual monomers within the dimer framework. The technique was implemented in an in-house program, which was used to carry out all the computations of the transfer integrals.

In particular, this technique is based on the construction of a two-state Hamiltonian in the basis of the Frontier molecular orbitals of the interacting monomers. Within the two-state approximation, the electronic structure of a dimer is thus described by the generalized secular equation

2 HC=SCE

where **H** and **S** are the Hamiltonian and overlap matrices, respectively, expressed in the basis of the monomer HOMOs (or LUMOs)

3 $$H=\left(\begin{array}{ll} e_{1} & J_{12}\\ J_{12} & e_{2} \end{array}\right)$$

and

4 $$S=\left(\begin{array}{ll} 1 & S_{12}\\ S_{12} & 1 \end{array}\right)$$

Since the monomer HOMOs (LUMOs) are not orthogonal, the Hamiltonian matrix elements *e*_*i*_ and *J*_12_ cannot be directly interpreted as site energies and transfer integral (i.e., electronic coupling), respectively. To recover this physical interpretation, the monomer orbitals are orthogonalized using the Löwdin symmetric orthogonalization procedure, which preserves the localized character of the monomer orbitals while introducing only a minimal delocalization. The effective Hamiltonian in the orthonormal basis is then

5 $$H^{eff}=\left(\begin{array}{ll} e_{1}^{eff} & J_{12}^{eff}\\ J_{12}^{eff} & e_{2}^{eff} \end{array}\right)$$

with

6 $$e_{1\left(2\right)}^{eff}=\frac{\left(e_{1}+e_{2}\right)-2J_{12}S_{12}\pm \left(e_{1}-e_{2}\right){\left(1-S_{12}^{2}\right)}^{1/2}}{2\left(1-S_{12}^{2}\right)}$$

and

7 $$J_{12}^{eff}=\frac{J_{12}-\frac{1}{2}\left(e_{1}+e_{2}\right)S_{12}}{\left(1-S_{12}^{2}\right)}$$

In the orthonormal basis, $e_{i}^{eff}$ and $J_{12}^{eff}$ recover their interpretation as effective site energies and transfer integral, respectively, and eq 2 reduces to the standard eigenvalue problem

8 $$H^{eff}C=CE$$

Within this framework, the energy splitting between the HOMO–1 and HOMO levels (or, equivalently, between the LUMO and LUMO+1 levels) of the dimer is given by

9 $$\Delta E_{12}=\sqrt{{\left(e_{1}^{eff}-e_{2}^{eff}\right)}^{2}+{\left(2J_{12}^{eff}\right)}^{2}}$$

which reduces to $2\left|J_{12}^{eff}\right|$ when the site energies $e_{1}^{eff}$ and $e_{2}^{eff}$ are identical.

To compute the charge transfer integrals using the approach outlined above, dimer geometries optimized at the DFT level were considered, employing the B3LYP-D3BJ and M06-2X functionals in combination with the 6-31G** and def2-SVPD basis sets. The latter was included to assess the effect of a larger and more flexible basis set incorporating diffuse functions on the calculated electronic couplings.

For the sake of completeness, it is worth mentioning that, throughout the present work, all baseline DFT calculations were carried out using the Gaussian 16 software package.^29^

### 2.2 Structural Descriptors

In this work, we considered different structural descriptors to compare the optimized geometries, namely the bond lengths, the torsional angle θ_CCNH_, the molecular planarity and the HOMA index.

The torsional angle θ_CCNH_ (in degrees) formed between the CNH planes in a pyrrole ring, was used to quantify the displacement of the hydrogen atom relative to the corresponding pyrrole ring. Its calculation was addressed using an in-house program (see Supporting Information).

The molecular planarity was described considering the atomic average absolute distance with respect to a reference plane. The optimized geometries were first centered at the origin and oriented parallel to the *YZ* plane using an in-house program. The molecular planarity (in Å) was afterward quantified as the average distance between the atoms and the *YZ* plane (thus calculated considering only the *X* coordinate).

The HOMA (Harmonic Oscillator Model of Aromaticity) index is a well-established descriptor of molecular aromaticity. For a given aromatic system, it is defined by the following equation^30,31^

10 $$HOMA=1-\frac{1}{N}\sum_{i}^{N}\alpha_{l}{\left(R_{i}-R_{l}\right)}^{2}$$

where *N* is the number of bonds and *R*_*i*_ the length of the *i*-th bond. The parameters $\alpha_{l}$ and $R_{l}$ depend on the nature of the atomic pair, l . Hereafter, we considered the values given in ref [.^32^ Thus, α_C–C_ = 257.7 Å^–2^ and *R*_C–C_ = 1.3880 Å for the C–C bond; α_C–S_ = 94.09 Å^–2^ and *R*_C–S_ = 1.6770 Å for the C–S bond; α_C–N_ = 93.52 Å^–2^ and *R*_C–S_ = 1.3340 Å for the C–N bond. These values were adopted in all our calculations, regardless of the choice of the functional used for geometry optimization.

### 2.3 Molecular Dynamics Simulations

Molecular dynamics (MD) simulations were performed on solvated and bulk systems. The optimized molecular structures of SU8, SN10H, and SN10O were used as starting points to build the input files for the MD simulations. The structure of SN10O was obtained from that of SN10H by replacing all hydrogen atoms with octyl (C_8_H_17_) chains in all-trans conformation.

For all molecules, intra- and intermolecular interactions were generated using the QForce program,^33^ using the extended tight-binding parametrization accessible via the XTB program.^34,35^ Some issues, encountered during the generation of the SN10O force field, were solved as described in the Supporting Information. The force field for the solvent, *ortho*-dichloro benzene (ODCB) was generated according to the same parametrization.

All MD simulations were carried out using the GROMACS program suite.^36,37^ The leapfrog algorithm was used to integrate the equations of motion, with a time step of 1 fs. The temperature was controlled by a velocity rescaling thermostat,^38^ with a coupling constant of 1.0 ps. The pressure was controlled via the Berendsen^39^ or the Parrinello–Rahman barostat,^40^ depending on the simulation (see Supporting Information).

Periodic boundary conditions were applied along all dimensions. Electrostatic interactions were treated using the Particle-Mesh-Ewald (PME) method^41^ with default settings (Fourier grid spacing of 0.12 nm and PME order of 4). The cutoff for nonbonded interactions was set at 1.4 nm. The force-switch modifier was not applied. Long-range dispersion corrections were enabled for energy and pressure. No constraints were applied to hydrogen atoms.

MD trajectories were collected every 50 ps and postprocessed to calculate the normalized radial distribution functions (RDF) reported in Subsection [Sec sec3.3]. All RDFs were calculated with respect to the molecular centers of mass. Specific details about the simulation protocols for solvated and bulk systems are available in the Supporting Information.

## 3 Results and Discussion

### 3.1 Structural and Electronic Properties

To compare the structural and electronic properties of sym-azathio circulenes with those of SU8, we optimized all molecular geometries at the B3LYP-D3BJ/6-31G** and M06-2X/6-31G** levels of theory.

Figure 1 shows the structures of SU8, SN8H, SN10H, and SN12H obtained at B3LYP-D3BJ/6-31G** level. Similar structures were obtained with functional M06-2X, except for some minor differences that will be discussed below.

For SN8H, the optimization results in a bowl-shaped geometry due to shorter carbon–nitrogen bonds (1.401 Å) compared to carbon–sulfur ones in SU8 (1.766 Å). Similar geometries have also been reported after complete replacement of sulfur with nitrogen atoms.^4^ SN10H can be thought as a derivative of C_20_S_10_,^42^ but, unlike the parent compound, its geometry is nearly planar (see below). Finally, SN12H is characterized by a crown-shaped structure. This outcome mirrors the one observed in sulflowers,^1^ where the increase in the molecular size also leads to increasingly strained geometries.

Table 1 collects the numerical values of selected structural and quantum mechanical descriptors for the investigated compounds. The average bond lengths enable a detailed comparison of the optimized geometries. In the sym-azathio circulenes, the inner-ring bonds of thiophene (C–C_inner*S*_) and pyrrole (C–C_inner*N*_) differ in length and were therefore treated separately. The bond lengths in SN8H closely resemble those reported for SU8. On going from SN8H to SN12H, all C–C bond lengths, including C–C_inner*N*_ and C–C_inner*S*_, show a slight increase, whereas the C–N bond distance exhibits the opposite trend, irrespective of the functional used. No significant differences are observed in the N–H bond lengths.

**Table 1:** Average Bond Lengths (in Å), Torsion Angle θ_CCNH_ (in Degrees), Planarity (in Å), HOMA Indices, and HOMO–LUMO Energy Gap (in eV) for SU8 (Where Applicable), SN8H, SN10H, and SN12H, Obtained From Calculations at Various DFT Levels^a^

	B3LYP-D3BJ/6-31G**				M06-2X/6-31G**			
	SU8	SN8H	SN10H	SN12H	SU8	SN8H	SN10H	SN12H*
C–C_inner*N*_	-	1.417	1.439	1.461	-	1.412	1.434	1.461
C–C_inner*S*_	1.423	1.425	1.447	1.474	1.422	1.425	1.447	1.480
C–C	1.382	1.385	1.398	1.408	1.373	1.375	1.387	1.395
C–S	1.766	1.794	1.758	1.750	1.755	1.783	1.750	1.738
C–N	-	1.401	1.380	1.368	-	1.392	1.373	1.360
N–H	-	1.008	1.006	1.006	-	1.006	1.005	1.005
θ_CCNH_ Min.	-	16.49	–18.58	–2.21	-	9.51	–0.19	–5.53
θ_CCNH_ Max.	-	16.70	18.66	2.07	-	9.74	1.59	5.62
planarity	0	0.402	0.0547	0.822	0	0.410	0.00169	0.734
HOMA_*N*_	-	0.786	0.770	0.640	-	0.825	0.837	0.693
HOMA_*S*_	0.634	0.417	0.561	0.377	0.686	0.485	0.618	0.419
HOMA_inner_	0.676	0.716	0.200	<0	0.700	0.646	0.280	<0
HOMO–LUMO gap	4.65	3.78	4.49	4.04	6.87	6.14	6.81	6.32

a Numerical differences are within the last reported digit or smaller. The value of HOMA_inner_ is unique and referred to the inner ring of each molecule. For θ_CCNH_, minimum and maximum values are reported.

The displacement of the hydrogen atom relative to the corresponding pyrrole ring was evaluated using the torsional angle θ_CCNH_, where a value of zero indicates an in-plane hydrogen. For out-of-plane hydrogens, the sign of θ_CCNH_, obtained from the calculation, can be positive or negative, depending on the position of the hydrogen with respect to the corresponding pyrrole ring (see Supporting Information for details).

Accordingly, in SN8H all hydrogen atoms adopt the same orientation, opposite to the direction of molecular curvature, regardless of the functional used. By contrast, in SN10H the hydrogen atoms alternate above and below the molecular plane with the B3LYP-D3BJ functional, whereas small deviations from planarity are observed for M06-2X. For SN12H an opposite trend can be observed.

To further investigate molecular planarity, we calculated the average distance between the atoms and a reference plane after molecular alignment (see Section 2). This descriptor can be expected to be exactly zero for perfectly planar molecules. Accordingly, our estimates predict SU8 to be perfectly planar, in line with other reports.^1,21^ Consistently with the above discussion, all sym-azathio circulenes exhibit deviations from planarity. Such deviations are modest for SN10H, and very small when the M06-2X functional is considered.

To evaluate the aromatic character of each compound, we computed the HOMA index (see Section 2 for details). The HOMA index is a simple and widely used measure of aromaticity for carbon-containing systems.^30,43^ A HOMA value of 1.0 corresponds to fully aromatic benzene, whereas a value of 0.0 represents a hypothetical Kekulé cyclohexatriene ring with complete bond-length alternation.

Hereafter, to provide a more comprehensive picture of the aromaticity distribution throughout the systems, we considered the HOMA index for thiophene (HOMA_*S*_) and pyrrole (HOMA_*N*_) rings. In addition, we extended the calculation to the ring made up by the innermost carbon atoms (HOMA_inner_).

As shown in Table 1, the majority of the aromatic character in these sym-azathio circulenes is localized within the pyrrole rings, regardless of the density functional employed. Indeed, the HOMA_*N*_ indices consistently exceed both the HOMA_*S*_ and HOMA_inner_ values. These indices exhibit magnitudes that depend on the molecular size, with trends that vary according to the chosen functional. Specifically, B3LYP-D3BJ yields a monotonic decrease of the HOMA_*N*_ index with molecular size, whereas the M06-2X calculations predict a maximum at SN10H.

Compared to SU8, the thiophene rings of sym-azathio circulenes exhibit a diminished aromatic character. For both the functionals considered, the HOMA_*S*_ values follow similar nonmonotonic trends, with peak values at SN10H.

The aromaticity of the inner rings decreases on going from SN8H to SN12H, due to the increase in C–C bond lengths (see above). The HOMA_inner_ indices of SN8H are close to those of SU8. The negative values obtained for SN12H result from the significant C–C bond length elongation compared to the reference value for benzene (1.388 Å).

Finally, we analyzed the HOMO–LUMO energy gap, which is often taken as a qualitative indicator of the lowest-energy electronic excitation.^44^ Our calculated values for SU8 are consistent with previous reports for both functionals.^4,21^ In particular, although the SU8 gap is larger than those obtained for many other organic semiconductors, this compound has nevertheless been employed as a p-type semiconductor in organic field-effect transistors (FETs).^7^ The energy gaps of the sym-azathio circulenes are comparable to those of SU8 and, interestingly, do not correlate with the aromatic character assessed by the HOMA index. The largest gap is found for SN10H and is only slightly smaller than that of SU8, despite arising from different HOMO and LUMO energy levels (see Supporting Information). For completeness, it is worth emphasizing that the calculations performed with the B3LYP-D3BJ and M06-2X functionals follow the same trend in the HOMO–LUMO gaps, although the M06-2X estimates are systematically higher than the B3LYP-D3BJ values, in agreement with previous studies.^21^

To evaluate the impact of exact Hartree–Fock exchange on our results, we replaced M06-2X with other functionals belonging to the M06 family, namely M06, M06-L, and M06-HF. The results, collected in the Supporting Information, confirm that M06-2X is a well-balanced choice, providing HOMO–LUMO gaps in between those of M06 (27% of Hartree–Fock exchange) and M06-HF (100% of Hartree–Fock exchange).

Finally, we repeated all the above calculations (including those with M06 functionals) replacing the 6-31G** with the 6-31+G** basis set. While the addition of diffuse functions systematically narrowed the HOMO–LUMO gaps, it had no significant impact on the overarching trends discussed above (see Supporting Information).

### 3.2 Charge Transport Properties

In the remainder of this paper, we shall focus on SN10H, which adopts a more planar geometry than SN8H and SN10H, and is thus expected to exhibit enhanced charge transport properties in the bulk. Here, these properties were evaluated at both the intra- and intermolecular levels.

#### 3.2.1 Intramolecular Electron Transport

To estimate the intramolecular charge (electron) transport properties, we followed the orbital rule proposed by Yoshizawa, based on the Green’s function analysis of molecular conductance in a metal-molecule-metal junction device.^22–24^ According to this approach, the HOMO–LUMO contribution to the 0-th order Green’s function ( $G_{rs}^{0}$ , see eq 1) provides a measure of electron transport efficiency through a single molecule where the atoms *r* and *s* are connected to external electrodes. If the product of the HOMO coefficients at the contact positions has the same sign as in the LUMO, the two terms in eq 1 cancel each other, leading to small $\left|G_{rs}^{0}\right|$ values. Conversely, the connections where these products exhibit different signs and large amplitudes (large $\left|G_{rs}^{0}\right|$ values) can be expected to significantly contribute to electron transport across the molecule.

According to this criterion, we reported in Table 2 the top three largest $\left|G_{rs}^{0}\right|$ values for each functional/basis set combination, sorted in decreasing order. The corresponding connections, labeled by capital letters, are reported in Figure 2. In order to obtain connections consistent with the molecular symmetry, degenerate or quasi-degenerate HOMO–1 and LUMO+1 orbital coefficients were also included in the calculations.

**Table 2:** Top Three Largest $\left|G_{rs}^{0}\right|$ Values Sorted in Decreasing Order for SU8 and SN10H, Calculated at the B3LYP-D3BJ/6-31G** and M06-2*X*/6-31G** Levels via Eq 1 ^b^

connection	B3LYP-D3BJ/6-31G**	connection	M06-2X/6-31G**
SU8			
A	2.143	A	1.388
B	1.748	B	1.170
C	1.742	C	1.133
SN10H			
D	0.916^a^	D	0.634
E	0.904^a^	E	0.588
F	0.882^a^	I	0.584

a For these connections the average value of $\left|G_{rs}^{0}\right|$ has been reported (see text).

b The connections, labeled by capital letters, are reported in Figure 2. Different labels were adopted to identify SU8 and SN10H connections. The full lists of connections and the corresponding $G_{rs}^{0}$ values are provided in the Supporting Information.

**Figure 2:**
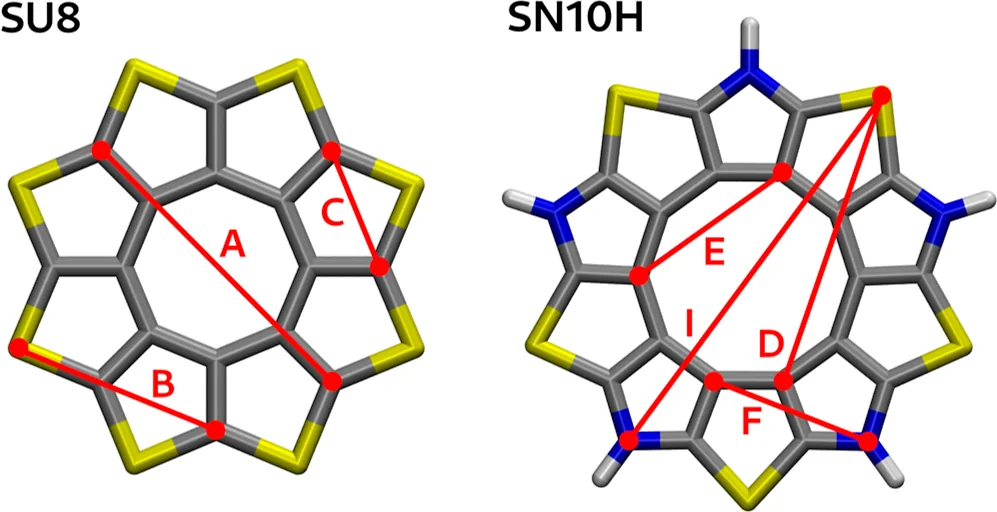
Intramolecular connections for SU8 and SN10H (S, yellow; C, gray; N, blue; H, white) as identified in Table 2. Symmetrically equivalent connections have been omitted for clarity.

For SU8, both functionals identify A, B, and C as the dominant transport connections. As illustrated in Figure 2, connection A involves the outermost carbon atoms; owing to the *D*_8*h*_ molecular symmetry of SU8, four such symmetry-equivalent connections exist. In contrast, connections B and C are shorter and yield comparable $\left|G_{rs}^{0}\right|$ values across both density functionals, featuring 16- and 8-fold multiplicities, respectively. Notably, the connection bridging the two most distant sulfur atoms (not shown) ranks fourth in this comparison, maintaining a non-negligible $G_{rs}^{0}$ value of 1.589.

For SN10H, the two functionals yielded contrasting results, primarily stemming from variations in their optimized molecular geometries. Calculations using the B3LYP-D3BJ/6-31G** geometry, which deviates slightly from perfect planarity (see above), produced marginally different $G_{rs}^{0}$ values for otherwise symmetrically equivalent connections. To simplify the discussion, these values were averaged in Table 2 and assigned to the same representative connection (D, E, or F in Figure 2). Connections D and E rank similarly for both functionals, despite M06-2X predicting a more planar geometry than B3LYP-D3BJ. Conversely, connection I, which couples the nitrogen and sulfur atoms on opposite sides of the molecule, ranks third on the M06-2X list but falls to sixth on the B3LYP-D3BJ list (see Supporting Information). Overall, our findings demonstrate that both SU8 and SN10H possess highly favorable short- and long-range connections for efficient electron transport.

#### 3.2.2 Intermolecular Charge Transport

The intermolecular electronic couplings (transfer integrals) governing charge transport were evaluated using the approach proposed by Valeev et al.^27,28^ already described in Section 2.1. Following this procedure, the Hamiltonian and overlap matrices were constructed using the monomer HOMOs to evaluate hole transfer integrals *J*_h_, whereas the corresponding LUMOs were employed to determine the electron transfer integrals *J*_e_. As mentioned above, these calculations were performed for each dimer geometry optimized at the corresponding level of theory, employing the B3LYP-D3BJ and M06-2X functionals in combination with the 6-31G** and def2-SVPD basis sets. The optimized dimer geometries obtained with the different functional/basis set combinations were found to be highly similar. Therefore, for the sake of clarity, only the structures optimized at the B3LYP-D3BJ/6-31G** level of theory are shown in Figure 3.

**Figure 3:**
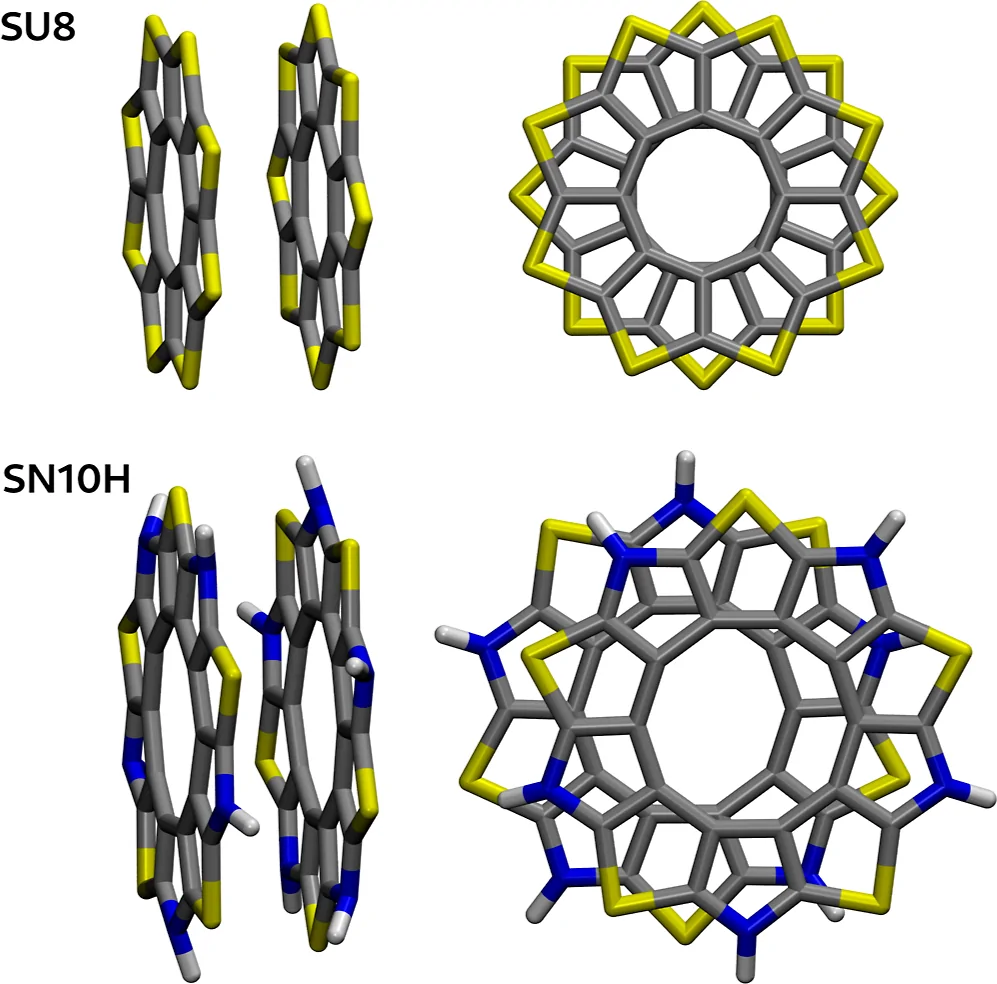
Side and front views of the SU8 and SN10H dimer geometries optimized at DFT level (B3LYP-D3BJ/6-31G**) (S, yellow; C, gray; N, blue; H, white).

Table 3 collects the transfer integrals obtained as described above. For SU8, our estimates of *J*_h_ align to those obtained by Mohakud et al.^5^ using a semiempirical approach on a stacked dimer geometry. It should be noted that the optimized dimer geometries used in this work differ from the packing arrangement of SU8 in the solid state.^1^ Therefore, although the interplanar distance in our dimers (between 3.37 and 3.44 Å) is close to the experimental value of 3.54 Å reported for the crystal structure,^1^ we may expect different results for crystal-like geometries.

**Table 3:** Hole (*J*_h_) and Electron (*J*_e_) Transfer Integrals (in meV) for SU8 and SN10H Computed at Various DFT Levels Using Optimized Neutral Dimer Geometries

	SU8 (D_8*d*_)		SN10H (*C*_2*h*_)	
*J*_h_	6-31G**	def2-SVPD	6-31G**	def2-SVPD
B3LYP-D3BJ	124.0	63.6	111.2	120.0
M06-2X	90.3	172.5	119.6	147.1

	SU8 (D_8*d*_)		SN10H (*C*_2*h*_)	
*J*_e_	6-31G**	def2-SVPD	6-31G**	def2-SVPD
B3LYP-D3BJ	81.6	64.8	73.4	83.9
M06-2X	121.8	175.1	55.9	71.4

The values of *J*_h_ for SN10H are comparable to those for SU8. We note that the SN10H molecules more closely approach each other than the SU8 ones in all our calculations. For example, for the B3LYP-D3BJ/6-31G** level of theory, the interplanar distance between SN10H molecules is 3.28 Å, whereas the one between SU8 ones is 3.44 Å. No significant differences are observed in the interplanar distances on going from 6 to 31G** to def2-SVPD for both molecules. This implies that the increase in the magnitude of the transfer integrals upon changing the basis set is probably caused by the inclusion of diffuse functions.

Inspection of the *J*_e_ values (see again Table 3) suggests that SU8 may behave as an ambipolar semiconductor. For the 6-31G** basis set, the magnitudes of *J*_e_ and *J*_h_ depend on the functional adopted. The value of *J*_h_ calculated with the B3LYP-D3BJ functional exceeds that obtained with the M06-2X functional. An opposite outcome is observed for *J*_e_. Conversely, closely matching values of *J*_e_ and *J*_h_ are obtained for the def2-SVPD basis set.

According to our results, SN10H better performs as hole than electron transporter. Finally, we note that the use of the def2-SVPD basis set leads to slightly larger *J*_h_ and *J*_e_ integrals than those obtained with the 6-31G** set of basis functions.

### 3.3 Solubility and Self-Assembling Properties

As a next step in our investigation, we replaced all hydrogens of SN10H with octyl chains to obtain its octyl derivative, hereafter abbreviated as SN10O. Compared to SN10H, SN10O can be expected to have similar transport properties, due to the marginal effect of alkyl chains on HOMO/LUMO energy levels,^45,46^ and enhanced solubility in organic solvents.

In order to test this hypothesis, we performed independent molecular dynamics (MD) simulations on SU8, SN10H and SN10O in *ortho*-dichloro benzene (ODCB). Suitable starting configurations were generated by evenly placing 64 solute molecules in a large simulation box, then solvated with 9000 ODCB molecules. After a short equilibration a production simulation (50 ns) was performed at constant temperature (300 K) and pressure (1 atm) for each system.

Figure 4 collects the simulation snapshots corresponding to the final configurations obtained at 50 ns. During the simulation, both SU8 and SN10H self-assembled in stacked molecular assemblies of variable size, comprising 2–6 molecules. Conversely, no such aggregates are visible for SN10O, whose molecules look more evenly dispersed.

**Figure 4:**
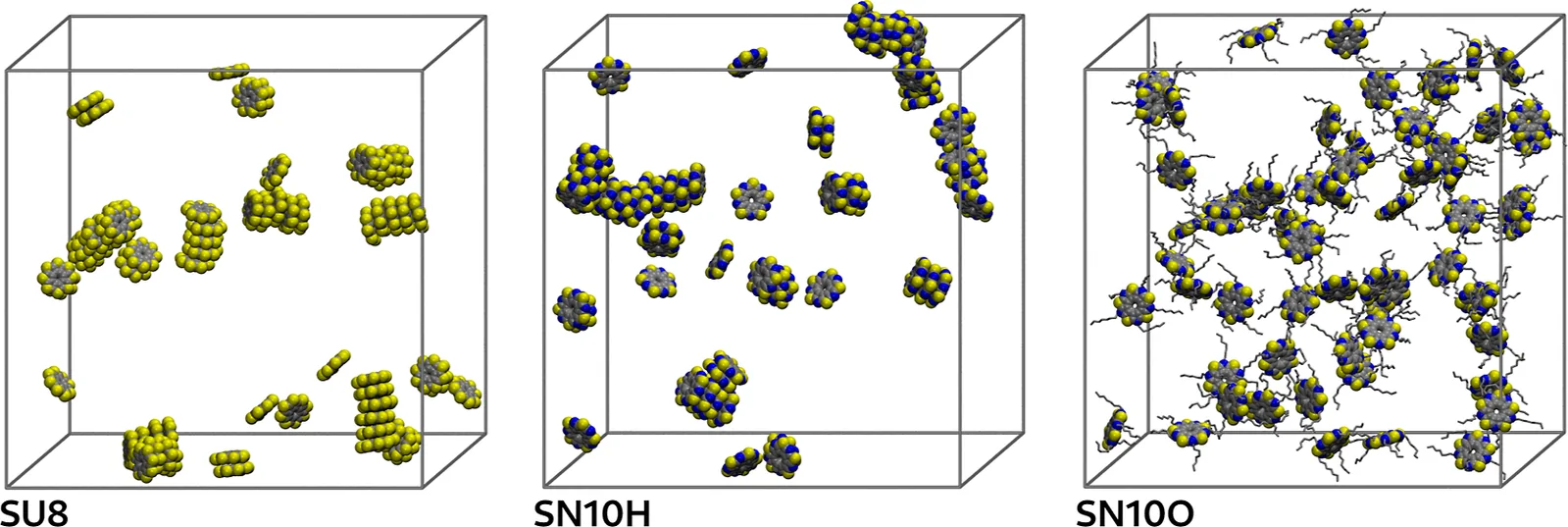
MD snapshots of SU8, SN10H, SN10O in ODCB at 50 ns. For better clarity solvent molecules and hydrogen atoms are omitted. (S, yellow; C, gray; N, blue).

To better quantify the above observations, we calculated for each system the normalized radial distribution function (RDF) with respect to the centers of mass of the solute molecules. As shown in Figure 5, the distributions of SU8 and SN10H are characterized by regular patterns of equally spaced peaks, corresponding to the spacings between consecutive neighbors within the molecular assemblies. For SU8, the average first neighbor distance is 3.84 Å, whereas, for SN10H, it is 3.72 Å. Both distances are comparable to those previously found for the corresponding dimers (i.e., 3.44 Å for SU8 and 3.28 Å for SN10H at B3LYP-D3BJ/6-31G** level, see above). On the contrary, the RDF of SN10O lacks any regular pattern. The first peak at 6.25 Å and the broad tail are consistent with the absence of tightly packed molecules and of a long-range order.

**Figure 5:**
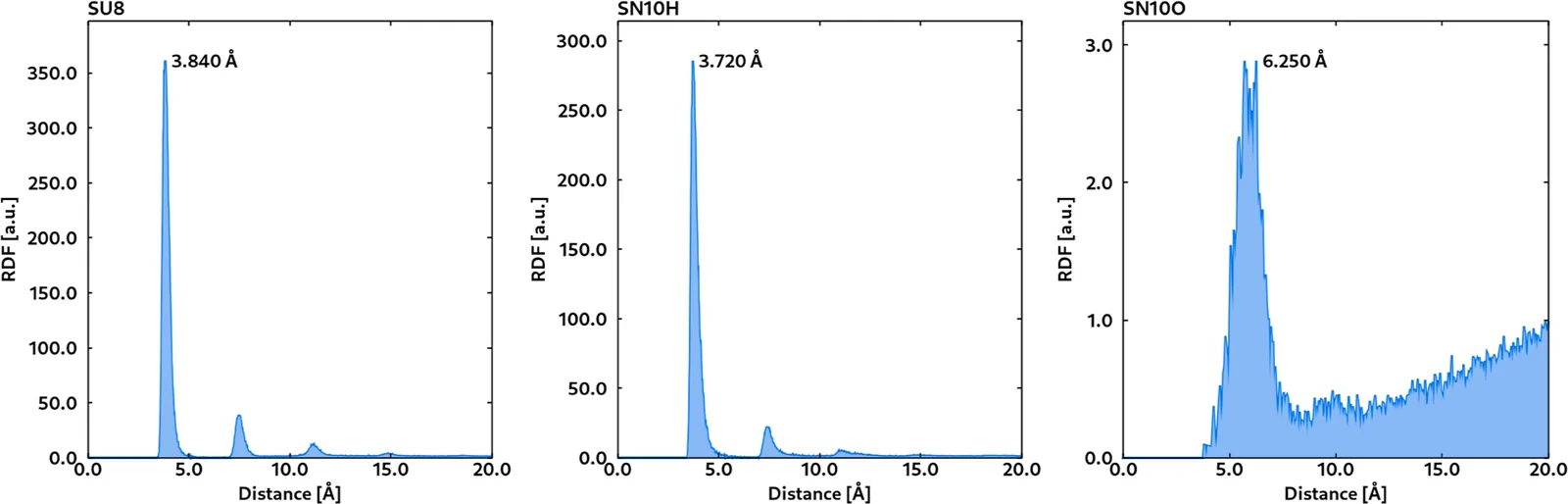
Normalized radial distribution functions for SU8, SN10H, and SN10O in ODCB.

The enhanced solubility of SN10O in ODCB, compared to SU8 and SN10H, demonstrates the effectiveness of side chain functionalization. Yet, it provides no information about the solid state organization of SN10O after solvent removal, which represents the final stage of solution processing. Experimentally, solvent evaporation takes place on time scales far beyond those achievable with atomistic simulations. Here, in order to gain some insights into SN10O self-assembling behavior, we simplified this process following a melt-and-cool route already exploited in our previous studies.^47,48^

We prepared a SN10O molten phase by heating a simulation box containing 540 SN10O molecules at 550 K and ambient pressure. The choice of this temperature was made after preliminary runs performed at different temperatures. The molten phase was gradually cooled down to 300 K. A last 15 ns simulation was finally performed to relax the final trajectory at the same temperature.

As shown in Figure 6, the final configuration obtained at 300 K for SN10O represents an amorphous phase, characterized by the presence of locally ordered domains, with clusters of π-stacked molecules, similar to those formed by SN10H.

**Figure 6:**
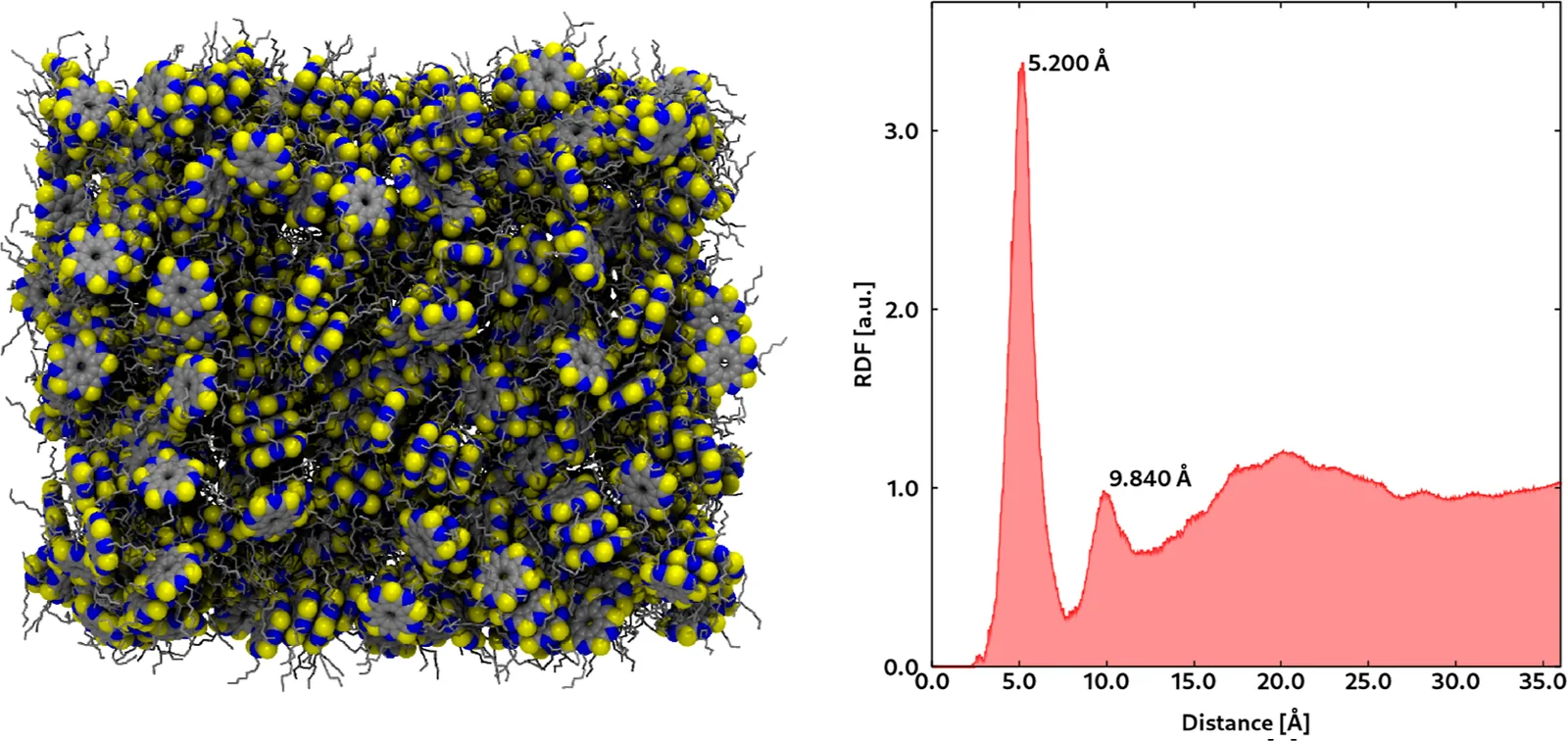
MD snapshot of the final configuration of bulk SN10O at 300 K (left); normalized radial distribution function calculated at the same temperature over the last MD run (right).

The corresponding RDF profile (see Figure 6) shows two main peaks at 5.20 and 9.84 Å associated with the first and second nearest neighbors shells. The former is consistent with the average separation between first neighbors in the molecular clusters also visible in the corresponding MD snapshot. The latter accounts for the contribution of clustered as well as isolated molecules. To evaluate the size of the molecular assemblies formed during the simulation, we calculated the average number of clustered molecules within a cutoff distance of 7.76 Å corresponding to the radius of the first coordination sphere (i.e., the first RDF minimum). The calculation was performed on the configuration shown in Figure 6 with an in-house program. Excluding single molecules from the calculation, we obtained an average cluster size of 4.24. This value is indicative of SN10O self-assembling behavior and greater than that obtained for SN10H in ODCB using the same protocol (i.e., 3.00), due to the larger size of the aggregates that may form in the bulk, compared to that of the aggregates in solution.

As mentioned above, alkyl side chains can be expected to have a little impact on SN10O HOMO/LUMO energy levels, compared to SN10H. Their presence may nevertheless introduce steric hindrance effects that affect molecular packing, and, in turn, charge transport properties. The magnitude of charge transfer integrals decays exponentially with the intermolecular distance.^28^ Based on the RDF reported in Figure 6, the values of *J*_e_ and *J*_h_ for SN10O should be smaller, on the average, than those reported in Table 3 for SN10H. This is not surprising, considering that our estimates for SN10H refer to gas-phase dimer structures at 0 K. Reasonably, the crystalline phases of SN10O would exhibit better charge transport properties than the amorphous structure that was reported here. At present, such phases are unknown and their determination is left to future experimental investigations.

## 4 Conclusions

In conclusion, in this work, we have explored the possibility to develop molecular precursors for solution-processable organic semiconductors starting from sulflowers, through the partial and symmetric replacement of sulfur with nitrogen. Our results suggest that sym-azathio circulenes possess structural and semiconducting properties comparable to those of SU8.

Among the compounds considered, SN10H represents the most promising candidate due to its planar structure and promising hole transport properties, as determined by means of the approach proposed by Valeev et al.^27^

This compound has been used as a starting point for the development of SN10O, which, according to our calculations, displays better solubility than SU8 and SN10O in ODCB. It is worth noting that SN10O is just one of the many derivatives of SN10H. Changing the alkyl side chain length may lead to compounds characterized by different solubilities. Alternative functionalizations of SN10H may also be explored with the aim of tuning its electronic properties.

Our results suggest that sym-azathio circulenes and their derivatives can be considered as promising synthetic targets in view of organic electronic applications. Recent advancements in the synthesis of hetero circulenes and related compounds^12,49–51^ suggest that their realization may soon become possible.

## Supplementary Material



## Notes

Details about the calculation of the θ_CCNH_ torsion. Additional views of SN8H and SN12H. HOMO and LUMO energy levels of monomers and dimers from DFT calculations. Molecular dynamics: simulation protocols for SU8, SN10H, and SN10O in ODCB and bulk SN10O (PDF)
