# Supplementary material for: In Silico Design of Molecular Precursors for Solution-Processable Organic Semiconductors: Structure and Electronic Properties of Sym-Azathio Circulenes
Source: ACS Omega. 2026 Sep 1;11(36):54187–95. doi: 10.1021/acsomega.6c05036 (PMC13584511; doi:10.1021/acsomega.6c05036)
Supplement: Supplementary file 1 [file ao6c05036_si_001.pdf]

Supporting Information for "In-silico Design of Molecular Precursors for Solution-processable Organic Semiconductors: Structure and Electronic Properties of Sym-azathio Circulenes"

Mosè Casalegno* and Alessandro Genoni

Department of Chemistry, Materials and Chemical Engineering "Giulio Natta", Politecnico di Milano, Via Bassini 6, 20133, Milano, Italy

E-mail: mose.casalegno@polimi.it

Contents

S1 Calculation of the θ_{CCNH} torsional angle **3**

S2 Additional views of SN8H and SN12H **4**

S3 HOMO and LUMO energy levels of monomers from DFT calculations **5**

S4 HOMO-LUMO contributions to the 0-th order Green's function (G^0_{rs}) for SU8 and SN10H from DFT calculations **7**

S5 HOMO and LUMO energy levels of dimers from DFT calculations **10**

S6 Force field generation for SN10O **11**

S7 MD simulation protocol for SU8, SN10H, and SN10O in ODCB **12**

S8 MD simulation protocol for bulk SN10O **13**

References **13**

S1 Calculation of the θ_{CCNH} torsional angle

As depicted in Figure S1.1 for SN8H, the torsional angle θ_{CCNH} was calculated as the angle formed by the two CNH planes highlighted in red. The effective value of θ_{CCNH} was obtained by appropriately shifting the calculated one by 180°, so that $\theta_{CCNH} = 0$ for in-plane hydrogens. The sign of the angle was the same obtained from the calculation, thus arbitrary but different for hydrogen placed above or below the corresponding pyrrole ring. In the main text we reported the minimum and maximum values obtained for each compound, respectively.

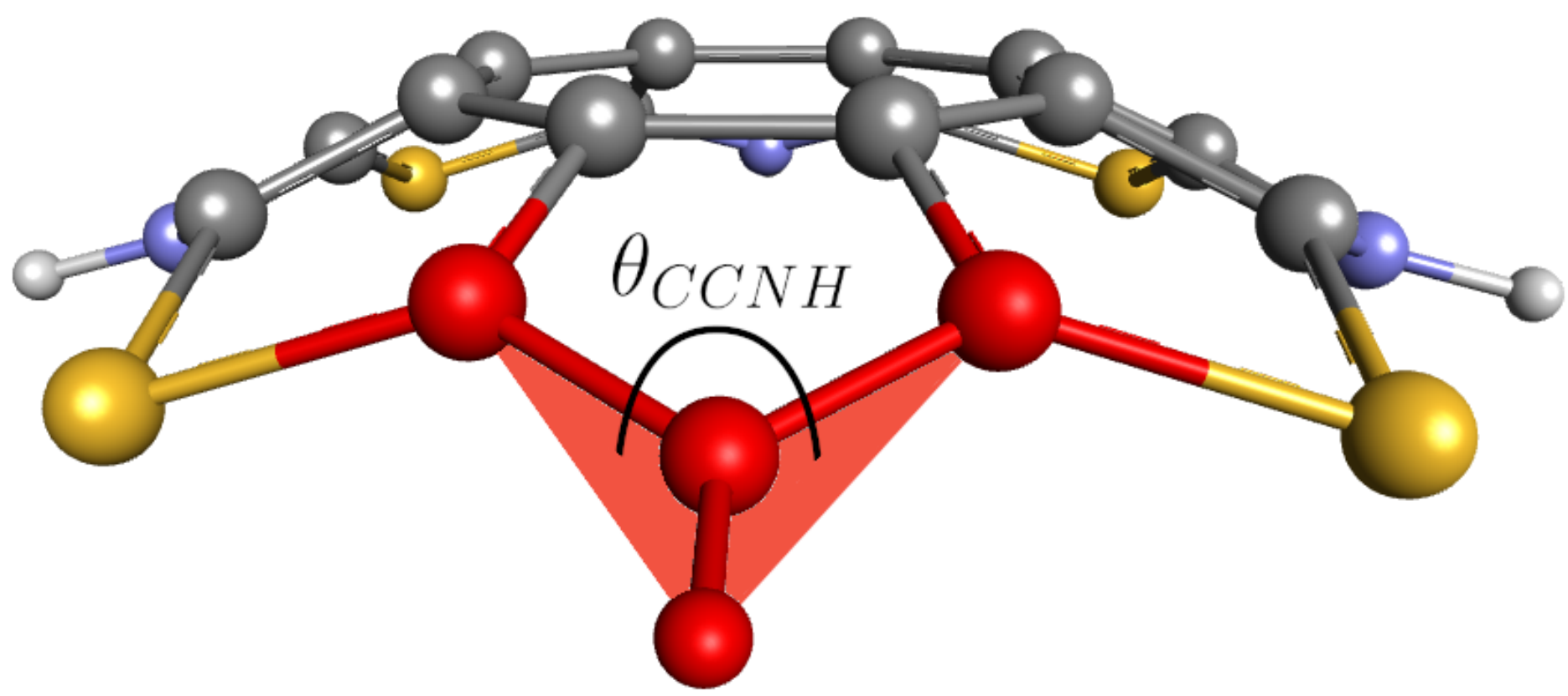

Figure S1.1: Sketch of SN8H illustrating the calculation of the torsional angle θ_{CCNH}. The atoms used in the calculation are highlighted in red.

S2 Additional views of SN8H and SN12H

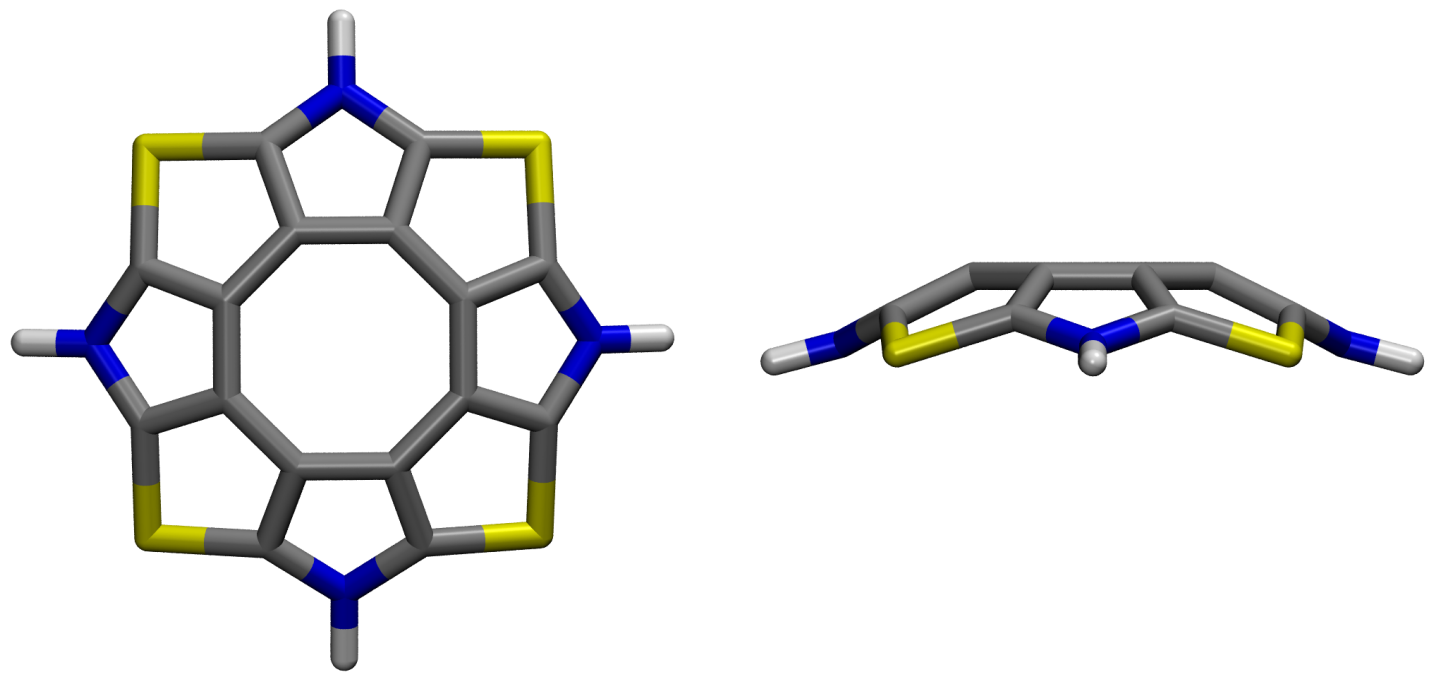

Figure S2.1: Views of SN8H (S, yellow; C, gray, N blue; H white). Geometry optimization was carried out at the DFT level (B3LYP-D3BJ/6-31G**).

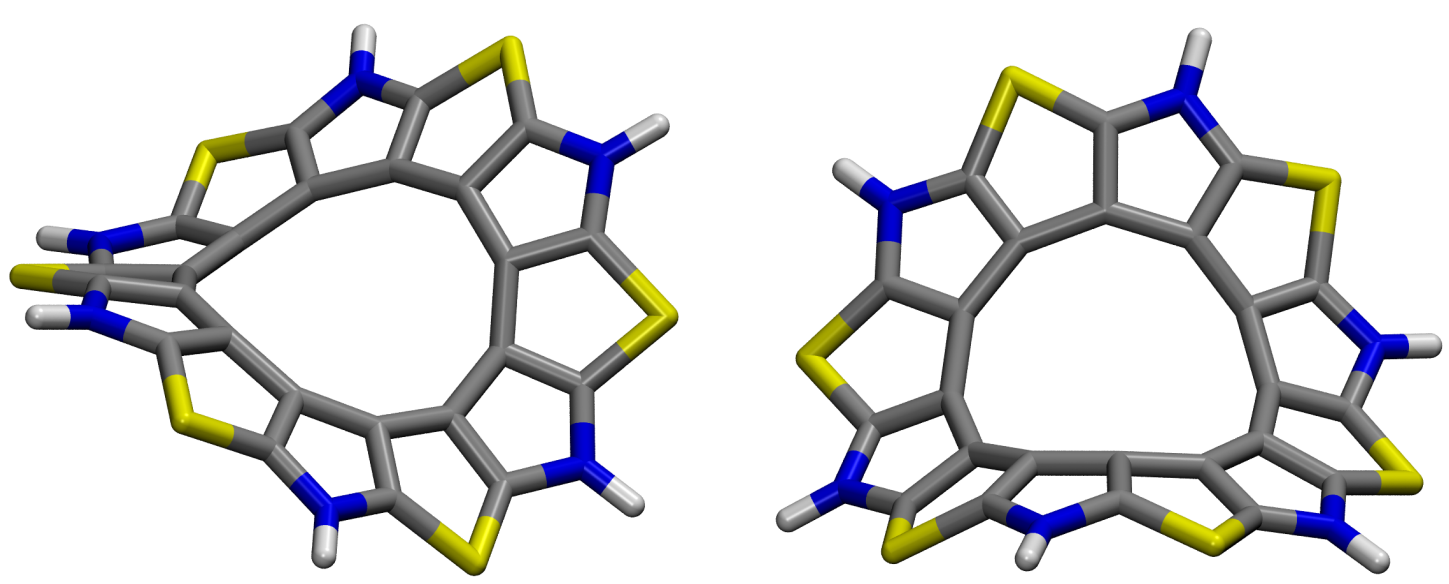

Figure S2.2: Views of SN12H (S, yellow; C, gray, N blue; H white). Geometry optimization was carried out at the DFT level (B3LYP-D3BJ/6-31G**).

S3 HOMO and LUMO energy levels of monomers from DFT calculations

Table S3.1: HOMO-1, HOMO, LUMO, LUMO+1 energy levels (in eV) for SU8, SN8H, SN10H, and SN12H, calculated using B3LYP-D3BJ and M06-2X functionals with basis set 6-31G**. All calcualtions refer to the corresponding optimized geometries. Molecular orbital indexes are also reported. *) The M06-2X/6-31G** values for SN12H refer to a structure with an imaginary frequency of -4.8453 cm^{-1}.

SU8	B3LYP-D3BJ	M06-2X
111 (HOMO-1)	-5.7312	-6.9892
112 (HOMO)	-5.7312	-6.9892
113 (LUMO)	-1.0778	-0.1154
114 (LUMO+1)	-1.0093	0.005714
SN8H	B3LYP-D3BJ	M06-2X
95 (HOMO-1)	-4.7824	-6.0173
96 (HOMO)	-4.7821	-6.0170
97 (LUMO)	-1.0057	0.1200
98 (LUMO+1)	-0.4147	0.7778
SN10H	B3LYP-D3BJ	M06-2X
119 (HOMO-1)	-4.5998	-5.8191
120 (HOMO)	-4.5881	-5.8189
121 (LUMO)	-0.09415	0.9929
122 (LUMO+1)	-0.08707	0.9932
SN12H*	B3LYP-D3BJ	M06-2X
143 (HOMO-1)	-4.3565	-5.6257
144 (HOMO)	-4.2939	-5.6058
145 (LUMO)	-0.2530	0.7157
146 (LUMO+1)	-0.0489	1.0291

Table S3.2: HOMO and LUMO energy levels (in eV) for SU8, SN8H, SN10H, and SN12H, calculated using B3LYP-D3BJ, M06-L, M06, M06-2X and M06-HF functionals with basis set 6-31G**. All calculations refer to the corresponding optimized geometries. Molecular orbital indexes are also reported.

SU8	B3LYP-D3BJ	M06-L	M06	M06-2X	M06-HF
112 (HOMO)	-5.7313	-5.1892	-5.9871	-6.9892	-8.6407
113 (LUMO)	-1.0778	-1.5540	-0.8640	-0.1154	1.0272
HOMO-LUMO gap	4.6534	3.6352	5.1231	6.8739	9.6679
SN8H	B3LYP-D3BJ	M06-L	M06	M06-2X	M06-HF
96 (HOMO)	-4.7821	-4.2267	-5.0853	-6.0170	-7.6867
97 (LUMO)	-1.0057	-1.6164	-0.8030	0.1200	1.5339
HOMO-LUMO gap	3.7764	2.6104	4.2823	6.1370	9.2206
SN10H	B3LYP-D3BJ	M06-L	M06	M06-2X	M06-HF
120 (HOMO)	-4.5881	-4.0847	-4.8937	-5.8175	-7.5076
121 (LUMO)	-0.0942	-0.4827	0.1472	0.9946	2.1141
HOMO-LUMO gap	4.4940	3.6020	5.0409	6.8121	9.6217
SN12H	B3LYP-D3BJ	M06-L	M06	M06-2X	M06-HF
144 (HOMO)	-4.3870	-3.8349	-4.6790	-5.6066	-7.2720
145 (LUMO)	-0.3401	-0.7848	-0.1298	0.7162	1.8213
HOMO-LUMO gap	4.0469	3.0501	4.5492	6.3228	9.0932

Table S3.3: HOMO and LUMO energy levels (in eV) for SU8, SN8H, SN10H, and SN12H, calculated using B3LYP-D3BJ, M06-L, M06, M06-2X and M06-HF functionals with basis set 6-31+G**. All calculations refer to the corresponding optimized geometries. Molecular orbital indexes are also reported.

SU8	B3LYP-D3BJ	M06-L	M06	M06-2X	M06-HF
112 (HOMO)	-5.8894	-5.2341	-6.0915	-7.1016	-8.7618
113 (LUMO)	-1.4746	-1.7413	-1.3176	-0.5238	0.1135
HOMO-LUMO gap	4.4148	3.4929	4.7740	6.5778	8.8753
SN8H	B3LYP-D3BJ	M06-L	M06	M06-2X	M06-HF
96 (HOMO)	-5.0575	-4.3778	-5.2989	-6.2273	-7.9076
97 (LUMO)	-1.5331	-1.8735	-1.4172	-0.5733	-0.0702
HOMO-LUMO gap	3.5244	2.5043	3.8817	5.6540	7.8374
SN10H	B3LYP-D3BJ	M06-L	M06	M06-2X	M06-HF
120 (HOMO)	-4.8668	-4.2254	-5.1103	-6.0132	-7.5071
121 (LUMO)	-0.7489	-0.8403	-1.0384	-0.1268	2.1149
HOMO-LUMO gap	4.1179	3.3851	4.0719	5.8864	9.6220
SN12H	B3LYP-D3BJ	M06-L	M06	M06-2X	M06-HF
144 (HOMO)	-4.6722	-3.9854	-4.8850	-5.8066	-7.4859
145 (LUMO)	-0.9140	-1.0757	-1.0596	-0.1992	0.0939
HOMO-LUMO gap	3.7582	2.9097	3.8254	5.6075	7.5797

S4 HOMO-LUMO contributions to the 0-th order Green's function (G^0_{rs}) for SU8 and SN10H from DFT calculations

Table S4.1: HOMO-LUMO contributions to the 0-th order Green's function (G^0_{rs}) for SU8 calculated at the DFT level, sorted by decreasing absolute value (see main text). Degenerate and quasi-degenerate molecular orbitals (HOMO-1 and LUMO+1) were included to obtain connections consistent with the molecular symmetry. The distance between the two atoms, d_{rs} in Å and the connection label are also reported. For atom numbering see Figure S4.1.

B3LYP-D3BJ/6-31G**					M06-2X/6-31G**				
Atom r	Atom s	G^0_{rs}	d_{rs}	Connection	Atom r	Atom s	G^0_{rs}	d_{rs}	Connection
9	13	-2.143	6.486	A	10	14	-1.388	6.462	A
11	15	-2.143	6.486	A	12	16	-1.388	6.462	A
10	14	-2.142	6.486	A	11	15	-1.388	6.462	A
12	16	-2.142	6.486	A	9	13	-1.388	6.462	A
11	18	1.748	4.248	B	10	20	1.170	4.228	B
9	24	1.748	4.248	B	12	22	1.170	4.228	B
13	20	1.748	4.248	B	14	24	1.170	4.228	B
15	22	1.748	4.248	B	16	18	1.170	4.228	B
10	20	1.748	4.248	B	14	21	1.170	4.228	B
14	24	1.748	4.248	B	12	19	1.170	4.228	B
12	22	1.748	4.248	B	10	17	1.170	4.228	B
16	18	1.748	4.248	B	16	23	1.170	4.228	B
11	21	1.748	4.248	B	9	19	1.170	4.228	B
15	17	1.748	4.248	B	15	17	1.170	4.228	B
9	19	1.748	4.248	B	13	23	1.170	4.228	B
13	23	1.748	4.248	B	11	21	1.170	4.228	B
10	17	1.748	4.248	B	9	24	1.170	4.228	B
14	21	1.748	4.248	B	15	22	1.170	4.228	B
12	19	1.748	4.248	B	11	18	1.170	4.228	B
16	23	1.748	4.248	B	13	20	1.170	4.228	B
11	12	-1.742	2.482	C	11	12	-1.133	2.473	C
13	14	-1.742	2.482	C	13	14	-1.133	2.473	C
9	10	-1.742	2.482	C	9	10	-1.133	2.473	C
15	16	-1.742	2.482	C	15	16	-1.133	2.473	C
14	15	-1.741	2.482	C	14	15	-1.133	2.473	C
10	11	-1.741	2.482	C	10	11	-1.133	2.473	C
9	16	-1.741	2.482	C	9	16	-1.133	2.473	C
12	13	-1.741	2.482	C	12	13	-1.133	2.473	C

Table S4.2: HOMO-LUMO contributions to the 0-th order Green's function (G^0_{rs}) for SN10H calculated at the DFT level, sorted by decreasing absolute value (see main text). Degenerate and quasi-degenerate molecular orbitals (HOMO-1 and LUMO+1) were included to obtain connections consistent with the molecular symmetry. The distance between the two atoms, d_{rs} in Å and the connection label are also reported. The connections G, H, J and K have not been reported nor discussed in the main text. For atom numbering see Figure S4.1.

B3LYP-D3BJ/6-31G**					M06-2X/6-31G**				
Atom r	Atom s	G^0_{rs}	d_{rs}	Connection	Atom r	Atom s	G^0_{rs}	d_{rs}	Connection
13	24	-0.938	6.464	D	12	21	-0.634	6.440	D
6	25	-0.938	6.464	D	19	27	-0.634	6.440	D
19	27	-0.937	6.463	D	1	12	-0.634	6.440	D
1	21	0.929	3.781	E	7	19	-0.634	6.440	D
6	15	-0.927	6.464	D	13	24	-0.634	6.440	D
9	30	-0.924	6.464	D	6	25	-0.634	6.440	D
4	24	-0.916	6.465	D	9	30	-0.634	6.440	D
1	28	0.916	3.569	E	6	15	-0.634	6.440	D
1	12	-0.91	6.463	D	18	30	-0.634	6.440	D
18	30	-0.909	6.465	D	4	24	-0.633	6.440	D
7	27	0.904	3.781	E	9	18	0.588	3.775	E
3	27	0.886	3.570	F	4	13	0.588	3.776	E
7	19	-0.884	6.464	D	15	25	0.588	3.776	E
1	6	0.878	3.921	G	1	21	0.587	3.776	E
21	28	0.877	3.568	F	7	27	0.587	3.775	E
24	27	0.876	3.921	G	3	19	-0.584	9.170	I
12	21	-0.874	6.464	D	12	28	-0.584	9.170	I
9	18	0.868	3.780	E	16	30	-0.584	9.170	I
4	18	-0.858	4.671	H	6	22	-0.584	9.170	I
9	25	-0.857	4.670	H	10	24	-0.584	9.170	I
6	22	-0.857	9.210	I	9	25	-0.575	4.662	J
10	24	-0.855	9.210	I	7	21	-0.575	4.661	J
3	19	-0.854	9.208	I	1	15	-0.575	4.662	J
4	13	0.854	3.780	E	13	27	-0.575	4.662	J
16	30	-0.854	9.209	I	4	18	-0.575	4.662	J
15	25	0.852	3.780	E	13	19	0.567	3.902	K
12	28	-0.849	9.207	I	19	21	0.566	3.902	K
13	27	-0.845	4.669	J	12	15	0.566	3.902	K

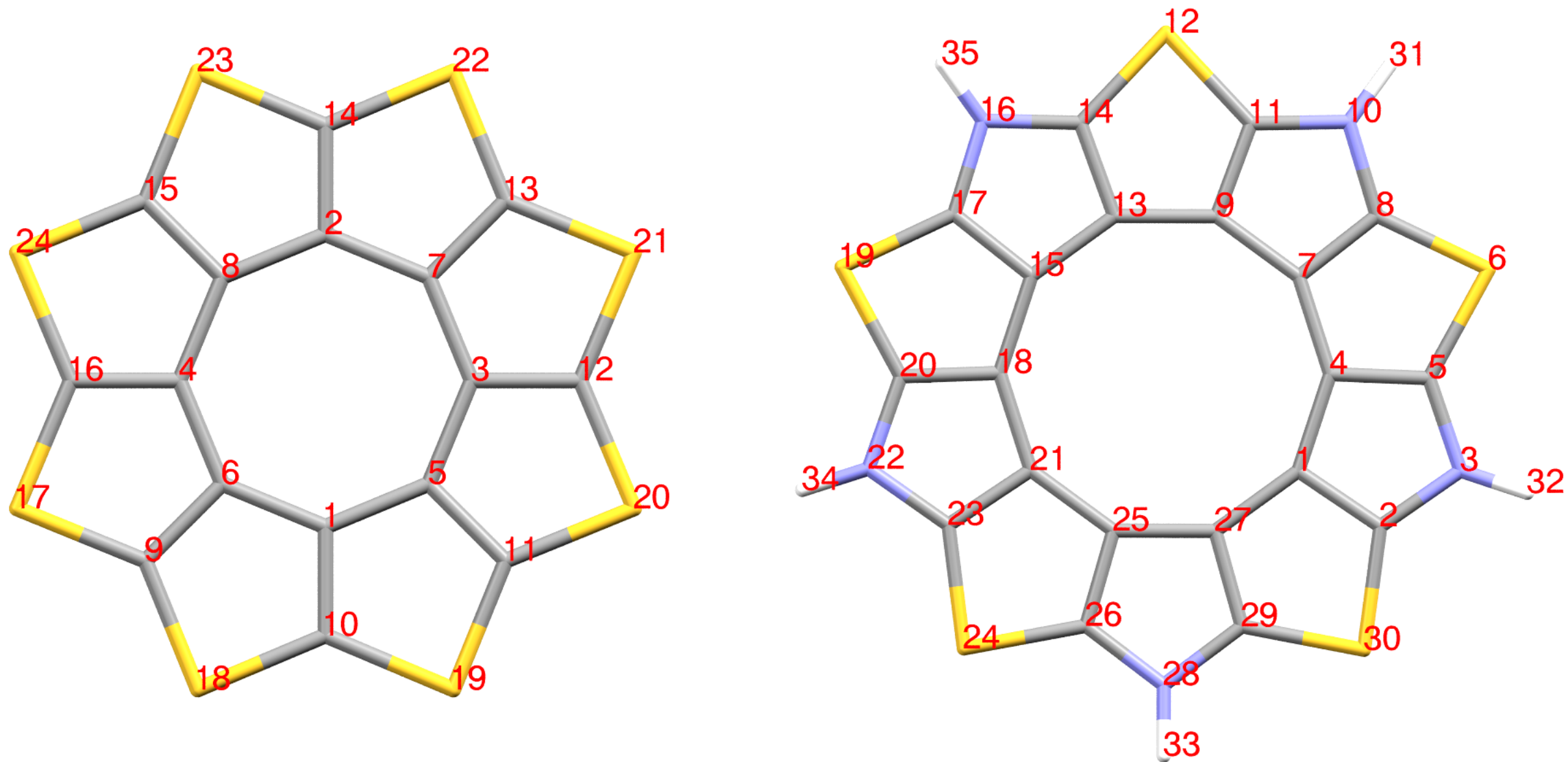

Figure S4.1: Structures and atom numbering of SU8 and SN10H. (S, yellow; C, gray; N, blue; H, white)

S5 HOMO and LUMO energy levels of dimers from DFT calculations

Table S5.1: HOMO and LUMO energy levels (in eV) for the SU8 and SN10H dimers calculated at DFT levels on optimized geometries. Molecular orbital indexes are also reported.

	B3LYP-D3BJ 6-31G**	M06-2X 6-31G**	B3LYP-D3BJ def2-SVPD	M06-2X def2-SVPD
SU8				
223 (HOMO-1)	-5.5356	-6.7974	-5.7410	-6.9337
224 (HOMO)	-5.5356	-6.7971	-5.7410	-6.9337
225 (LUMO)	-1.3091	-0.4501	-1.7845	-1.0601
226 (LUMO+1)	-1.1069	-0.1630	-1.5265	-0.6422
SN10H				
239 (HOMO-1)	-4.4401	-5.5508	-4.6757	-5.8221
240 (HOMO)	-4.3571	-5.5484	-4.5734	-5.6877
241 (LUMO)	-0.2244	0.9069	-0.7965	-0.2092
242 (LUMO+1)	-0.1107	0.9997	-0.6702	-0.13878

S6 Force field generation for SN10O

As mentioned in the Materials and Methods section, some issues were encountered upon submitting the SN10O molecule to the QForce program, due to the steric hindrance of the octyl chains, which hampered the generation of consistent force field parameter sets over independent attempts. To solve this issue, we adopted an intermediate structure (see Figure S6.1), characterized by a single chain. The dihedral parameters were then used to model all chains in SN10O.

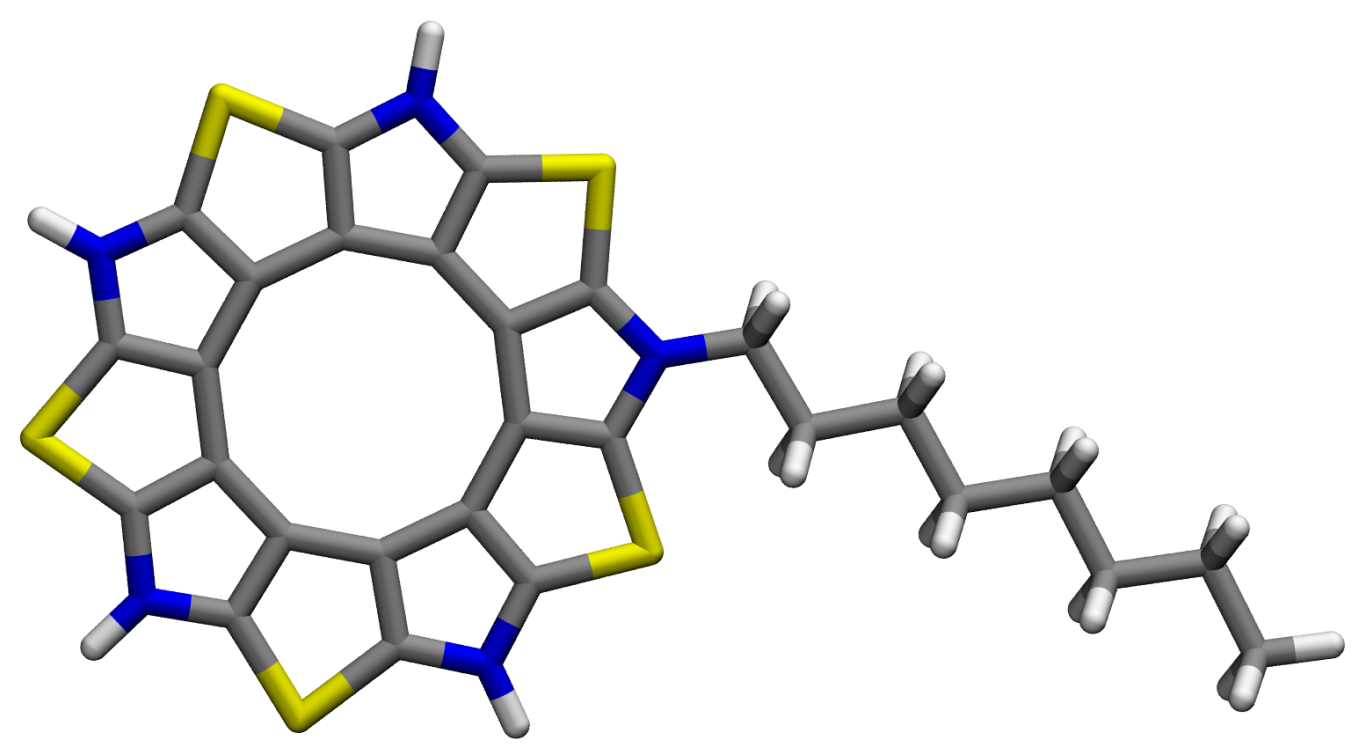

Figure S6.1: Intermediate structure of SN10O with a single alkyl chain used in force field development.

S7 MD simulation protocol for SU8, SN10H, and SN10O in ODCB

For each compound (SU8, SN10H, and SN10O), a starting MD input file was generated by evenly placing 64 molecules in large cubic simulation boxes. The size of each box was chosen large enough to accommodate 9000 ODCB molecules. After solvation, energy minimization was performed on all model systems to prevent atomic clashes. Then, for each system a 50-ns simulations was performed at constant temperature (300 K) and pressure (1 atm). In all simulations, the box shape was kept orthorhombic, with the box lengths allowed to vary and the angles fixed at 90°. The pressure was controlled with the Berendsen barostat. The coupling constant was set at 5.0 ps and the isothermal compressibility at 4.5×10^{-5} atm^{-1}. Attempts at using the Parrinello-Rahman barostat[1] resulted in large and uncontrolled box deformations, regardless of the value of the pressure coupling constant. Work is underway to refine the simulation protocol and perform the simulation using this barostat. Table S7.1 provides some useful data about the simulated systems, including the starting and final box side lengths and systems densities.

Table S7.1: Data for the simulated systems: starting and final box side lengths and systems density (averaged over 50 ns).

System	Starting box side [nm]	Final box side [nm]	Average density [g/L]
SU8	12.20	11.87	1338.21(6)
SN10H	12.20	11.88	1338.05(4)
SN10O	12.50	12.51	1318.36(5)

S8 MD simulation protocol for bulk SN10O

The starting configuration for bulk simulations was generated by randomly placing 540 SN10O molecules in a large simulation box. A first simulation (5 ns) was performed at ambient temperature (300 K) and high pressure (1000 atm) to quickly adjust the box size. The resulting configuration was then relaxed at high temperature (550 K) and ambient pressure (1 atm) for 10 ns, in order to obtain a molten phase. In both the above runs the pressure was controlled with the Berendsen barostat, with the same settings defined above. The molten phase was then cooled down from 550 to 300 K at ambient pressure by gradually decreasing the temperature at a rate of 4.16 K/ns. The final structure was maintained at 300 K for 15 ns. The overall simulation time was 75 ns. The pressure was controlled with the Parrinello-Rahman barostat, setting the coupling constant at 50.0 ps and the isothermal compressibility at 4.5×10^{-5} atm^{-1}. In all simulations, the box shape was kept orthorhombic, with the box lengths allowed to vary and the angles fixed at 90°. It is worth noting that, in the absence of information on the crystal phases of SN10O, we chose the temperature of 550 K based on preliminary test simulations performed at different temperatures.

References

(1) Parrinello, M.; Rahman, A. Polymorphic transitions in single crystals: A new molecular dynamics method. *Journal of Applied Physics* **1981**, *52*, 7182–7190.
